# Musculoskeletal Disorders in Chronic Obstructive Airway Diseases: A Neglected Clinical Entity

**DOI:** 10.31138/mjr.32.2.118

**Published:** 2021-06-30

**Authors:** C. Mohan Rao, Pratima Singh, Debashis Maikap, Prasanta Padhan

**Affiliations:** 1 Department of Pulmonary Medicine, Kalinga Institute of Medical Sciences, KIIT University, Bhubaneswar, Odisha, India; 2 Department of Clinical Immunology and Rheumatology, Kalinga Institute of Medical Sciences, KIIT University, Bhubaneswar, Odisha, India

**Keywords:** COPD, Skeletal muscle, osteoporosis

## Abstract

Chronic obstructive pulmonary disease (COPD) is a lung disease that can affect various extra-pulmonary organs; one being the musculoskeletal system. Skeletal muscle dysfunction and osteoporosis are two important musculoskeletal disorders that have an impact on the quality of life in COPD patients in terms of morbidity and mortality. Treatment related adverse effects of COPD such as steroid-induced myopathy and osteoporosis are well recognised. Other comorbidities like sarcopenia, cardiovascular disease, metabolic diseases (diabetes mellitus, obesity, and thyroid diseases), chronic kidney disease, sleep apnoea, anaemia, and depression are also noted, which can contribute to impaired health status, increased healthcare utilisation, and even mortality. As well, it has been shown that autoimmunity and autoimmune rheumatic diseases (AIRDs) are linked to COPD. In this mini-review, we intend to give an overview of different types of musculoskeletal disorders associated with COPD.

## INTRODUCTION

Chronic obstructive pulmonary disease (COPD) is a clinical syndrome characterised by chronic respiratory symptoms, structural pulmonary abnormalities (airway disease, emphysema, or both), lung function impairment (primarily airflow limitation that is poorly reversible), or any combination of these.^1^ It is the third leading cause of death worldwide - COPD led to 3.2 million deaths in 2017, a toll that is expected to reach 4.4 million yearly by 2040.^2,3^ With a worldwide disease prevalence of 10.1% and 6% all deaths, it is a major cause of chronic morbidity, where years of life lost prematurely has increased 13.2% between 2007 and 2017.

The relentless decline in lung function that characterises COPD is associated with progressive symptoms and functional impairment. Often, COPD patients have susceptibility to respiratory exacerbations, most commonly through infection. Exacerbations are responsible for much of the morbidity and mortality.^4^ By definition, a COPD exacerbation means an increase in dyspnoea, cough or sputum production, with or without symptoms of upper respiratory tract infection.

Small airway diseases present with cough and dyspnoea. Often these symptoms may be the initial presentation of the illness, even preceding systemic manifestations. Symptoms may also commonly coexist with rheumatic autoimmune diseases (AIRDs). Thus, COPD is an umbrella term for various pathological entities because of multiple causes resulting in airway obstruction that is not fully reversible. Notably, almost every COPD patient is diagnosed with one or more comorbid conditions, where illnesses such as cardiovascular disease, depression and lung cancer have received the most attention. However, musculoskeletal symptoms remain under detected in routine clinical evaluations.

**Figure 1.**
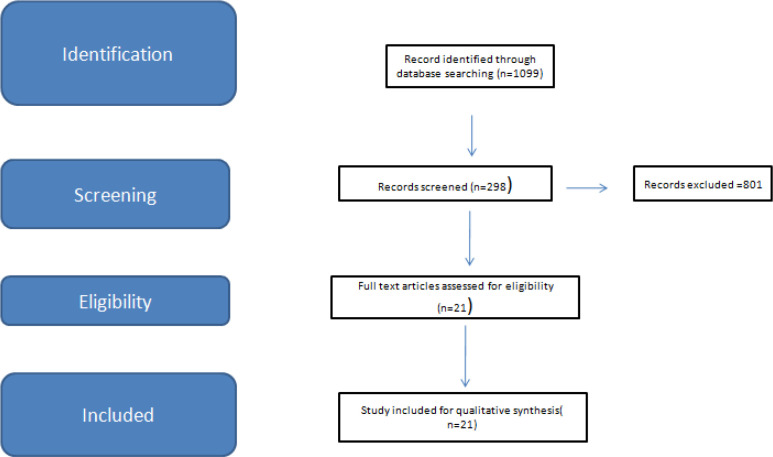
Flow diagram of the Systematic search.

There is an increased prevalence of COPD in autoimmune diseases, which suggests systemic autoimmunity could be a risk factor for its development.^5^ It is hypothesized that autoimmunity is causally related to the development of COPD, likely as a result of inflammation triggered by environmental factors like smoking.

Besides lung abnormalities, COPD can impact other organ systems leading to comorbidities.^6–8^ It is unclear whether these associations are a consequence of shared risk factors such as smoking, atmospheric pollution or low physical activity, or whether COPD itself is a genuine causal factor. The major extrapulmonary effects include skeletal muscle dysfunction and osteoporosis. Others include anaemia, cardiovascular diseases, lung cancer, and diabetes mellitus.

## MATERIAL AND METHODS

The authors performed a systematic search of patients with musculoskeletal dysfunction and COPD in PubMed, Scopus from January 1980 until September 2020. Keywords in the search were “Musculoskeletal System”[MeSH] OR “Musculoskeletal Abnormalities”[MeSH] OR “Musculoskeletal Pain”[MeSH] OR “Musculoskeletal Diseases”[MeSH]) AND “Pulmonary Disease, Chronic Obstructive”[MeSH]. We did not exclude any paper by language or publication date. After screening, we selected 21 records for review as per author discretion.

### Skeletal muscle dysfunction

Skeletal muscle dysfunction is known to occur in COPD patients, and can be characterised by reduced muscle strength, reduced muscle endurance, and muscle fatigue.^9^ There are 2 types of fibres in skeletal muscle, and Type I are aerobically utilised while type II are anaerobically utilised. There is a reduced proportion of type I fibres and an increase in type II fibres. Muscle mass decreases and type IIa fibers tend to convert to type IIb. Deconditioning is probably very important in muscle dysfunction in COPD. Other mechanisms that may be of varying importance in muscle dysfunction include chronic hypercapnia and/or hypoxia, nutritional depletion, steroid usage, oxidative stress, smoking, malnutrition, and immobilization, which all eventually lead to accelerated intracellular protein degradation - the hallmark of muscle atrophy. It occurs via two primary mechanisms: the ubiquitin–proteasome and lysosomal pathways, which coexist in COPD and operate in a coordinated manner.^10^

The prevalence of skeletal muscle weakness in COPD is about 32%. Muscle weakness is often seen in the lower limb muscles of patients with COPD. Quadriceps muscle weakness is an early feature due to physical inactivity, inflammation, and oxidative stress.^11^ It is common in all stages of COPD in both men and women and is a predictor of mortality.^12^ During acute exacerbations in COPD; lower limb weakness is more severe in patients with cachexia.^13^ Typically, upper limb muscles in patients with COPD are spared. Similarly, there is a trend towards a higher prevalence of skeletal muscle weakness with increased COPD disease severity (GOLD stages).^14^ In the lower limb muscles, several adaptations develop with COPD, which include a type I to type II muscle fibre shift with reduced oxidative capacity, increased glycolytic capacity, muscle fibre atrophy, loss of muscle mass, and reduced capillary density.^9^

### Systemic inflammation

Systemic inflammation has been reported in patients with severe COPD with muscle wasting. Serum levels of tumour necrosis factor (TNF)-α, its receptors, interleukin (IL)-1β, IL-6, IL-8, IL-18, and acute phase reactants are found to be raised in COPD.^15^ In the Wust et al. study, patients with acute COPD exacerbations that have increased serum levels of IL-8 were surprisingly found to be negatively associated with quadriceps weakness. In contrast, IL-6 levels remained the same and TNF α was undetectable.^16^

Muscle wasting is present in 6% to 21% of patients of COPD. Hypoxia has been postulated for the initiation and progression of muscle wasting^17^ - ultimately having a significant impact on the quality of life. It can even be associated with premature death.

### Sarcopenia

Sarcopenia is defined as loss of muscle function. The amount of muscle dysfunction is often associated with the prevalence of chronic diseases such as type 2 diabetes mellitus, chronic heart failure, chronic kidney disease - COPD is no exception. Its prevalence in COPD is around 15% to 25%. Sarcopenia is associated with loss of mobility, osteoporosis, poor quality of life, increased falls, hospitalisation, and even death. Losing muscle mass and function lead to decreases in physical activity and energy consumption leading to an increase in body weight.^17^ It may ultimately result in quadriceps weakness due to mechanical unloading of the muscle and muscle wasting, which can be associated with poor muscle endurance.^13^

The mortality rate for those with sarcopenia is found to be 2.99 times higher in males and 3.22 times higher in female than a normal group as described in a study by Korean scientists, where 500 elderly patients over 65 years were followed for six years.^18^ It is imperative to diagnose sarcopenia in COPD cases with a low quality of life. The European working group on sarcopenia in older people (EWGSOP) presented a European consensus on the definition and diagnosis of sarcopenia in 2010 which has been updated in 2018 by EWGSOP2.^19^ These definitions should be utilised in the identification of sarcopenia in patients with chronic illnesses such as COPD.

Cigarette smoke is a significant contributor to skeletal muscle weakness, and it has been shown to exert adverse effects on bone. It also causes reduced skeletal muscle strength and physical performance.^13^

Corticosteroids are often used in patients with COPD to reduce pulmonary symptoms and to treat exacerbations. Chronic steroid use is associated with steroid myopathy, proximal muscle weakness due to muscle fibre atrophy, and increased risk of fall and fracture. Because oral steroids may have an adverse effect on skeletal muscle function, their use should be avoided whenever possible.^13^ Other factors like aging, hypogonadism, vitamin D deficiency, and undernutrition may also have a role in skeletal muscle dysfunction.

Sarcopenia can be prevented with progressive resistance exercise, aerobic exercises, adequate nutritional support with a protein-rich diet, and vitamin D. Potential therapies include exercise training, oxygen supplementation, nutritional repletion, and administration of anabolic hormones. Testosterone replacement therapy, nutritional intervention and vitamin D correction may also help in some patients. Exercise, especially fall prevention and balance training, may improve muscle strength and stability.

### Metabolic bone diseases

Metabolic bone diseases are usually caused by a disturbance in minerals (such as calcium or phosphorus) and vitamin D metabolism, resulting in low bone mass or bone structure affecting bone strength, with osteoporosis being the most common. Osteoporosis is a silent systemic skeletal disease characterised by microarchitectural reduction of bone tissue leading to a low bone mass, increased bone fragility and thereby increased fracture risk.^20^ The fracture risk depends on bone strength, which is determined by bone mineral density (BMD) (determined by peak bone mass and amount of bone loss) and bone quality (a function of bone architecture, turnover, damage accumulation and mineralization).^21^ It is generally accepted that BMD accounts for approximately 70% of bone strength.^22^ In COPD patients, bone quality is impaired (low trabecular bone score with increased cortical porosity) and is associated with low bone turnover. Due to a lack of clinical tools for precise evaluation of bone quality except for a few markers for bone turnover, a diagnosis of osteoporosis has been based on BMD measured by dual-energy X-ray absorptiometry (DXA). Bone mineral density is expressed in the standard deviation of means, the T and Z scores. The T score is a standard deviation compared to a young adult sex-matched control population. The Z score is a standard deviation compared to an age- and sex-matched control population. As per WHO, osteoporosis is defined as a T score of less than –2.5 and osteopenia is defined as T score between –1 and –2.5.^23^ One standard deviation reduction in the BMD increases the fracture risk by 1.5–3x.^22^ Low BMD and low bone quality is a predictor of insufficiency fractures.^23^ COPD patients may also develop osteomalacia, which should not be confused with osteoporosis, as both the cause and treatment differ despite their coexistence. In osteoporosis, the mineral-to-collagen ratio is within the normal range, whereas in osteomalacia, the proportion of mineral composition is reduced relative to organic material content. The leading cause of osteomalacia is vitamin D deficiency, which may be seen with increased incidence in housebound COPD patients due to a reduced cutaneous production of Vitamin D. It may also occur with nutritional deficiency, malabsorption, chronic liver disease, long term anticonvulsive therapy, and phosphate deficiency.^24^ The clinical features of osteomalacia include vague musculoskeletal pain and muscle weakness. In early stages, osteomalacia may be misdiagnosed as a variety of musculoskeletal diseases including osteopenia and osteoporosis, and for early diagnosis high degree of suspicion of osteomalacia is necessary. Bone densitometry may detect osteoporosis in up to 70% of patients with osteomalacia.^25^

COPD-associated osteoporosis is significantly under-recognized, and hence undertreated. According to recent epidemiological data, osteoporosis is highly prevalent among COPD patients.^23^ In COPD patients, the prevalence of osteoporosis is assumed to be two- to fivefold higher than that in age-matched subject without airflow obstruction.^26^ Osteoporosis leads to increased occurrence of fractures with disability and mortality, which adds to the economic burden of the disease. In a retrospective study of 234 male subjects, a high prevalence of osteoporosis was noted secondary to COPD.^27^ Moreover, osteoporosis-associated fractures may further deteriorate pulmonary function and impair activities of daily life (ADL), quality of life (QOL), respiratory function, and possibly prognosis.^28^ Thus, the two diseases can form a vicious cycle, causing a significant health burden in these patients.

Osteoporosis is common in both male and female patients with COPD. Approximately one third of all COPD patients have osteoporosis and another one third have osteopenia in a study where the mean age of subjects was 63 years with FEV1 of 47 %.^29,30^ This in turn may result in vertebral compression fractures and hip fractures.^34–36^ The prevalence of morphometric fractures could even be as high as 24%–79% based on age, sex, and COPD severity.^23^

Osteoporosis in COPD has a complex aetiology, with various factors contributing to its pathogenesis. Some of these factors are lung damage, reduced physical activities, steroid therapy and natural changes due to aging including hypogonadism. When fractures occur as a complication of osteoporosis, the quality of life of such patients that are usually already movement-restricted due to lung disease is further reduced. In general, body mass index (BMI) is a determinant of BMD and fracture risk, and the BMI-associated fracture risk is mostly dependent on BMD.^23^ Unfortunately, weight loss is frequently found in COPD, particularly at advanced stages, and therefore is associated with increased fractures and poor prognosis.^23^ Additional risk factors such as smoking, alcohol, low vitamin D, and chronic kidney disease also play a role in osteoporosis in COPD.

Glucocorticoid-induced osteoporosis (GIO) develops in a dose-dependent manner and occurs even at small doses. They have both direct adverse effects on bone by suppressed bone formation and indirect effect because of muscle weakening and atrophy. Oral glucocorticoids cause a decrease in vascular endothelial growth factor, leading to decreased skeletal angiogenesis, bone hydration, and strength.^16,29^ These effects are both dose-dependent and duration-dependent. An episodic five-day course of glucocorticoids administered during acute exacerbations as per GOLD guideline recommendations are relatively devoid of these adverse effects, and inhaled corticosteroid (ICS) use have not been shown to aggravate the bone mineral loss in COPD patients.^16^ However, according to a recent meta-analysis including 16 RCTs with 17,513 subjects and seven observational studies with 69,000 subjects, ICS are found to be associated with significant fracture risk (OR=1.27 for RCTs and 1.21 for observational studies). Overall effects of ICS depend on the balance between its anti-inflammatory effects and fracture risk, caused due to its systemic effects.^32^

Osteoporosis is an important area of consideration for therapeutic intervention. Exercise training, particularly weight bearing and strengthening exercises may be effective for maintaining skeletal health to prevent fracture in elderly patients, besides smoking cessation, good nutrition, calcium, and vitamin D supplementation.^33^ Efforts should be made to detect BMD early in COPD patients and prompt addition of bisphosphonate or teriparatide/denosumab, as therapy should be done in the presence of osteoporosis. In COPD patients, gastroesophageal reflux disease is frequently seen and often marked in acute exacerbations. In such cases, other bisphosphonates such as intravenous zoledronate or subcutaneous denosumab or teriparatide can be used. It is worthwhile to differentiate osteoporosis from osteomalacia as inappropriate use of bisphosphonates in patients with undiagnosed osteomalacia in COPD may be associated with focal demineralization bone defects.

#### Vitamin D insufficiency/deficiency

According to the Endocrine Society Clinical Practice Guideline, vitamin D deficiency and insufficiency are defined as 25-Hydroxy Vitamin D levels below 20 and 20–30 ng/mL, respectively.^34^ There is a high prevalence Vitamin D deficiency in COPD, which may be due to decreased sunlight exposure, poor diet and smoking.^23^ Eventually it can lead to poor calcium absorption from gut, reduced bone calcification, secondary hyperparathyroidism with high bone turnover causing bone loss and increased fracture risk. Skeletal muscle dysfunction and osteomalacia are seen as a consequence of vitamin D deficiency, which can lead to muscle weakness and falling, increasing fracture risk in a BMD-independent manner.

Hypovitaminosis D is related to COPD can result in increased susceptibility to infection.^35^ Emerging evidence indicates that vitamin D-mediated innate immunity, chiefly by improved expression of the human antimicrobial peptide, is significant in defence against respiratory tract pathogens. Deficiency in 25-hydroxyvitamin D is related to the emerging risk of infections containing influenza, tuberculosis, and pneumonia.^36^ Control of respiratory tract infection through correction of vitamin D level may result in a better outcome in patients with COPD.^37^ Vitamin D supplementation may reduce exacerbations in COPD patients with severe vitamin D deficiency.^38,39^

### COPD and Autoimmune Rheumatic Diseases (AIRDs)

Although there is no strong evidence about the association between AIRD and COPD, a few possible shared mechanisms common to both are citrullination, autoimmunity, and systemic inflammation. However, smoking may be a confounding factor, as it is a well-established risk factor for both COPD and AIRDs, along with diet and obesity. The presence of chronic autoreactive systemic inflammation increasingly appears to be most strongly associated with emphysema in smokers.^40^ Autoimmune diseases are characterised by activated T helper cells (Th1 and Th17) and their respective canonical cytokines (INF-γ, IL-17A and TNF).^48,49^ This immune response which is essential for AIRDs model; a signature of autoimmunity, has been demonstrated in patients with emphysema.^40^ There is also increased prevalence of organ-specific autoimmune diseases in patients with COPD not associated with smoking^50^; namely, older women with lymphopenia, positive auto-antibodies. Those patients having unexplained chronic cough and lymphocytic airway inflammation are eight times more likely to have autoimmune disease, and some develop COPD irrespective of smoking status.

Among various autoimmune rheumatic disorders, the most robust evidence for the development of COPD exists in patients (particularly women) diagnosed with rheumatoid arthritis (RA) when compared to patients without any AIRDs.^41^ In particular, anti-citrullinated peptide antibodies (ACPAs), a hallmark of RA, is consistently found increased in tobacco- or non-tobacco-induced COPD. However, it is important to remember that that seropositivity (defined as positive rheumatoid factor or anti-cyclic citrullinated peptide antibodies) does not have a significant effect on COPD risk as reported by Sparks et al.^42^ It is also noted that AIRDs can predispose to COPD by chronic systemic inflammation with or without local respiratory lesions. In addition to cigarette smoking, AIRDs may itself be a determining factor for increased COPD incidence and/or facilitate a shortened time course of development of COPD.^43^

There is some evidence to support the contention that there is increased acquired autoimmunity in COPD:

- Both helper (CD4+) and cytotoxic T lymphocytes (CD8+) accumulate in the lung parenchyma of patients with COPD^44,45^;
- Increased B lymphocytes in bronchus-associated lymphoid tissue (BALT) which has been shown to be significantly in smokers and in patients with COPD^46^;
- Smoking is associated with an expansion of the population of antigen presenting cells on the epithelial surface of the lower respiratory tract;
- Increased prevalence of antinuclear antibody titres in patients with COPD compared with smokers with normal lung function.^46^

It has been demonstrated by one study that COPD is associated with the production of autoantibodies against a broad spectrum of self-antigens, and immune reactivity to lung.^47^ Increased numbers of activated antigen-presenting cells (specifically Th1 and Th17 cells) have been associated with smoke-induced lung inflammation. The production of the canonical cytokines from these cells, such as IFN-γ and IL-17, correlate with disease severity.^40^ Similarly, other inflammatory joint disorders, such as psoriatic arthritis, ankylosing spondylitis, and connective tissue disorders, such as Systemic lupus erythematosus, primary Sjogren’s disease, and Systemic sclerosis, are seen more in COPD than the general population and are associated with a greater risk of mortality, hospital-isations, and emergency visits.^43^ Anti-nuclear antibodies (ANA) and anti-tissue (AT) antibodies have been found in approximately in one-third of patients with COPD.^51^

Importantly, in COPD/AIRD coexistence, clinicians should be aware of the possibility of worsened long-term health outcomes and act promptly so that suffering can be minimised by interventions such as smoking cessation.^43^

## CONCLUSION

Thus, in COPD, many definitive types of musculoskeletal consequences may be witnessed through systemic effects or as an aftermath of diverse immunological musculoskeletal disorders. It is now understood that COPD is more of a syndrome than a single disease. The use of pulmonary rehabilitation has a negative impact on comorbidities. Alternatively, a multidisciplinary patient-centred approach along with a rational use of inhaled/systemic glucocorticoids with lower doses and systemic anti-inflammatory agents has paved the way for optimum effective treatment of COPD with a consequential reduction of comorbidities.

The concept of COPD as one of a group of coexisting (multi-morbidity) disorders, including AIRDs, deserves attention because treatment based on the common pathobiological processes may have a simultaneously beneficial effect on a variety of target organs.
